# Evaluating the competence of the primary vector, *Culex tritaeniorhynchus*, and the invasive mosquito species, *Aedes japonicus japonicus*, in transmitting three Japanese encephalitis virus genotypes

**DOI:** 10.1371/journal.pntd.0008986

**Published:** 2020-12-28

**Authors:** Astri Nur Faizah, Daisuke Kobayashi, Michael Amoa-Bosompem, Yukiko Higa, Yoshio Tsuda, Kentaro Itokawa, Kozue Miura, Kazuhiro Hirayama, Kyoko Sawabe, Haruhiko Isawa

**Affiliations:** 1 Laboratory of Veterinary Public Health, Graduate School of Agricultural and Life Sciences, The University of Tokyo, Bunkyo, Tokyo, Japan; 2 Department of Medical Entomology, National Institute of Infectious Diseases, Shinjuku, Tokyo, Japan; 3 Department of Environmental Parasitology, Tokyo Medical and Dental University, Bunkyo, Tokyo, Japan; 4 Pathogen Genomics Center, National Institute of Infectious Diseases, Shinjuku, Tokyo, Japan; Stanford University, UNITED STATES

## Abstract

Japanese encephalitis virus (JEV) is maintained in an enzootic cycle between swine, water birds, and mosquitoes. JEV has circulated indigenously in Asia, with *Culex tritaeniorhynchus* as the primary vector. In some areas where the primary vector is scarce or absent, sporadic cases of Japanese encephalitis have been reported, with *Aedes japonicus japonicus* presumed to have the potential as a secondary vector. As one of the world’s most invasive culicid species, *Ae*. *j*. *japonicus* carries a considerable health risk for spreading diseases to wider areas, including Europe and North America. Thus, evaluation of its competency as a JEV vector, particularly in a native population, will be essential in preventing potential disease spread. In this study, the two mosquito species’ vector competence in transmitting three JEV genotypes (I, III, and V) was assessed, with *Cx*. *tritaeniorhynchus* serving as a point of reference. The mosquitoes were virus-fed and the infection rate (IR), dissemination rate (DR), and transmission rate (TR) evaluated individually by either RT-qPCR or focus forming assay. Results showed striking differences between the two species, with IR of 95% (261/274) and 9% (16/177) in *Cx*. *tritaeniorhynchus* and *Ae*. *j*. *japonicus*, respectively. Both mosquitoes were susceptible to all three JEV genotypes with significant differences in IR and mean viral titer. Results confirm the primary vector’s competence, but the fact that JEV was able to establish in *Ae*. *j*. *japonicus* is of public health significance, and with 2%–16% transmission rate it has the potential to successfully transmit JEV to the next host. This may explain the human cases and infrequent detection in primary vector-free areas. Importantly, *Ae*. *j*. *japonicus* could be a relevant vector spreading the disease into new areas, indicating the need for security measures in areas where the mosquito is distributed or where it may be introduced.

## Author summary

It is widely known that not all mosquito species serve as medically-important virus’ vectors. Even among the vectors, there are variations in their ability to transmit one virus or the other. Nonetheless, while the known primary vectors’ transmission efficiency is evident, the importance of confirming the ability of other potential vectors is indispensable when it comes to the threat of disease introduction to new areas. Japanese encephalitis is a mosquito-borne disease mainly circulating in Asia. However, pockets of infection have been detected outside this endemic area, where the primary mosquito vector (a Culex species) is almost nonexistent. One possibility is that the causal virus is carried by the invasive mosquito species (an Aedes species), originating from Asia, confirmed to adapt well in Europe and North America. Thus, a thorough evaluation will be needed to determine the competence of both the Culex and Aedes species to transmit Japanese encephalitis virus (JEV). Here, the authors found that although the difference in the transmission efficiency between the Culex and Aedes was significant, their competence as vector is evident. This research provides evidence of the possible relevance of the Aedes species in the distribution and spread of JE into new areas.

## Introduction

Japanese encephalitis virus (JEV) is a mosquito-borne flavivirus belonging to the family *Flaviviridae*. Clinical cases of JEV were first reported in humans in Japan in 1871 [1,2]. The virus is postulated to be maintained in an enzootic cycle between swine, water birds, and mosquitoes in the genus *Culex* [3,4]. For almost a century, it has circulated indigenously in most parts of Asia and the Pacific [2]. Despite being asymptomatic in most human infections, a fraction of infected people, below 1%, develop JEV-related encephalitis symptom with about a 30% of fatality rate in children while 30–50% survivors develop neurological sequelae [1].

JEV has a positive-sense single-stranded RNA genome that contains a large open reading frame (ORF) flanked by 5′ and 3′ untranslated regions (UTRs). The ORF encodes a polyprotein consisting of structural proteins [capsid (C), membrane (prM/M), and envelope (E)] and nonstructural (NS) proteins NS1, NS2A, NS2B, NS3, NS4A, NS4B, and NS5 [5].

**Fig 1:**
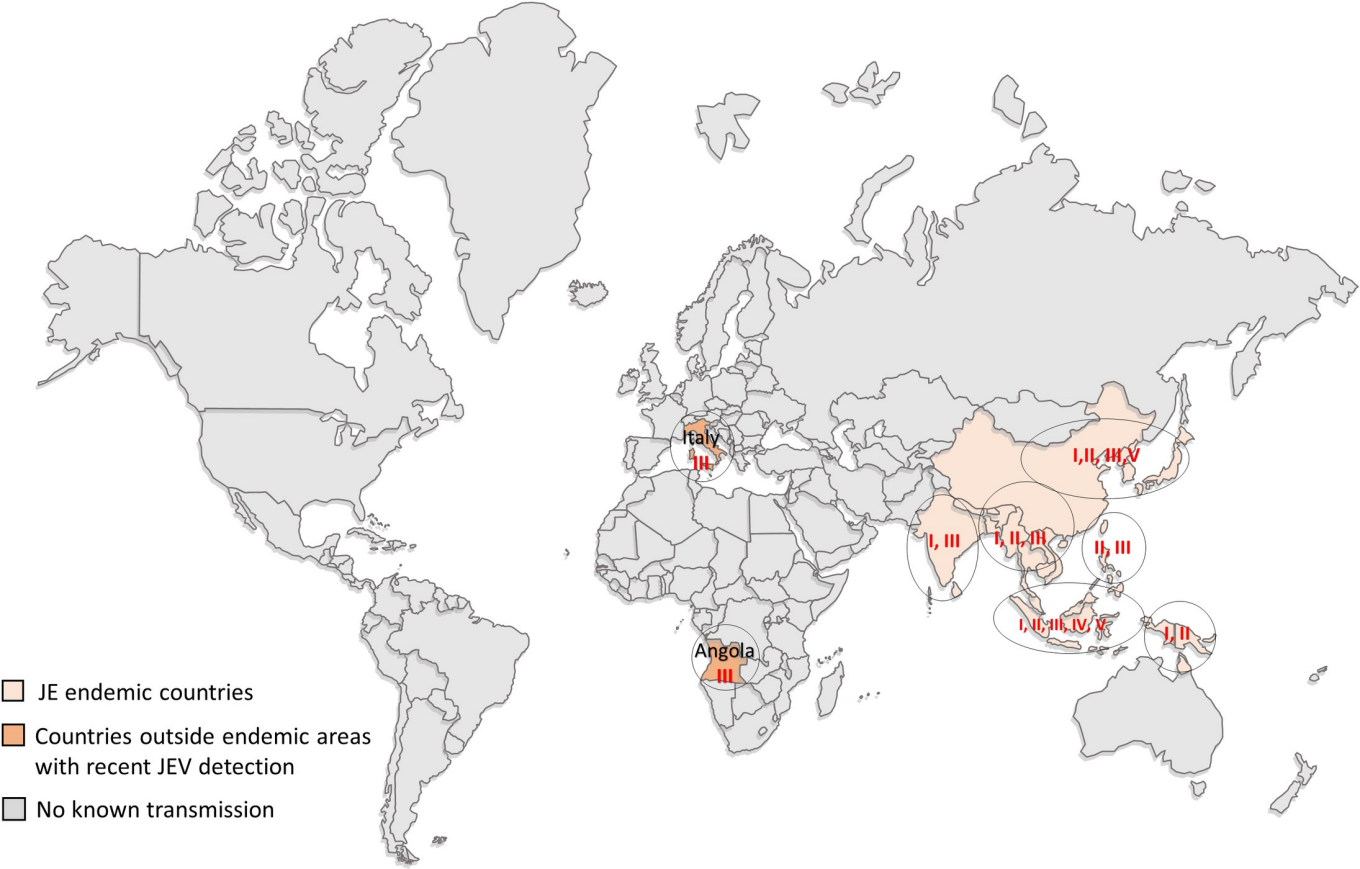
Geographic distribution of JEV. World geographic distribution of JEV genotypes (I to V) with colors representing endemic countries, mostly in Asia, and two countries with recent JEV GIII detection outside its endemic areas.

JEV is genetically classified into five genotypes (GI–GV) based on its E gene sequences (Fig 1); the current dominant genotype in East Asia is GI, having displaced GIII about 30 years ago [6]. The other three genotypes (GII, GIV, and GV), on the other hand, seem to be confined to the Indo-Malay region [7,8]. Although JEV GV used to be a rare lineage with relatively limited distribution, it is now considered to be re-emerging following recent detection in mosquitoes in China and South Korea [9,10] and in a JE-vaccinated human patient in South Korea [11]. Accordingly, as well as the two dominant genotypes (GI and GIII), JEV GV was included in this study because it is considered a rising threat in Japan.

**Fig 2:**
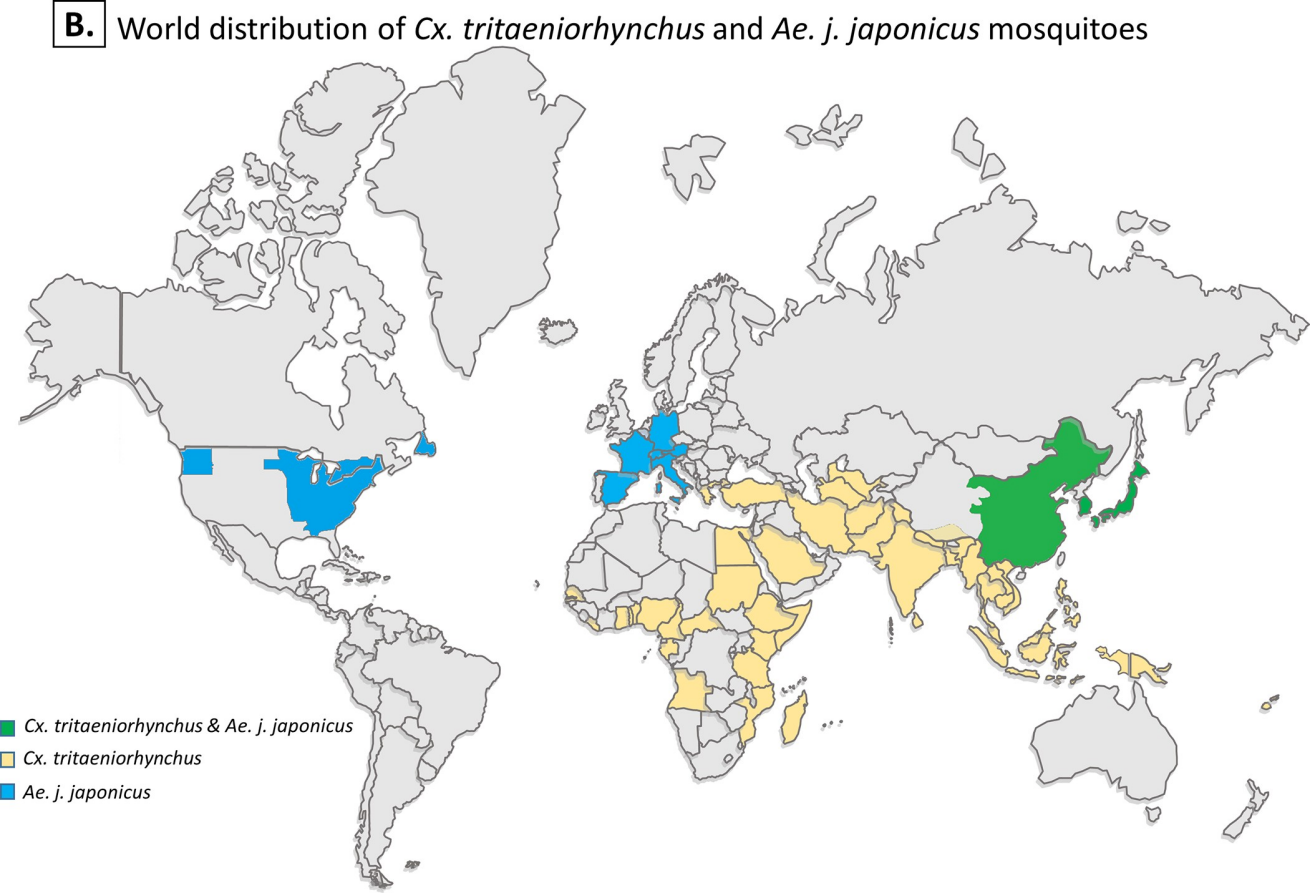
Geographic distribution of two mosquito species used in this study. World distribution of *Cx*. *tritaeniorhynchus* and *Ae*. *j*. *japonicus* mosquitoes. Colors representing areas, states, or countries, with single mosquito species (in yellow or blue) and the combination of both (in green). In addition, the two mosquito species have putatively been collected in parts of southeastern Russia, which is not shown on the current map.

The primary vector of JEV in most endemic areas in Asia, including Japan, is *Culex vishnui* subgroup, especially *Cx*. *tritaeniorhynchus* Giles, 1901 [12–14] (Fig 2). However, in some areas (e.g., rice paddies and pig husbandry areas) in which the primary vector does not thrive due to sub-optimal ecological conditions, cases of JE have been observed despite *Cx*. *tritaeniorhynchus* being almost absent. JEV infections in humans in Gangwon-do, South Korea [15], and in swine in Hokkaido, Japan, for example [16], raise concerns about the possibility of other mosquito species acting as secondary vectors, such as *Cx*. *pipiens pallens*, *Cx*. *quinquefasciatus*, *Cx*. *modestus ficalbi*, *Cx*. *orientalis*, *Cx*. *bitaeniorhynchus*, *Armigeres subalbatus*, and *Aedes* (syn. *Finlaya*) *japonicus japonicus* [17,18].

*Ae*. *j*. *japonicus* (Theobald, 1901), also known as the Asian bush mosquito or Asian rock pool mosquito, is listed among the most invasive culicid species, second only to *Ae*. *aegypti* [19] Its invasive nature means *Ae*. *j*. *japonicus* brings considerable health risks via introduction and spread of diseases to wider areas. With an original distribution range in East Asia, there are reports of *Ae*. *j*. *japonicus* infesting other continents (Fig 2).

*Ae*. *j*. *japonicus* was reported in 1979 by Tanaka et al. [20] as a species originated from temperate regions in Asia including Japan, Korea, southern China and Taiwan, and southeastern Russia. Detections of *Ae*. *j*. *japonicus* being outside its native range was first reported in New Zealand in 1993 [21], in the United States in 1998 [22], and in Europe in 2000 [23]. Monitoring efforts of the invasive mosquito species in Europe has revealed the successful establishment by multiple introductions of the colony in ten countries, including Germany, Belgium, Switzerland, Italy, and Austria, mostly Central Europe [24,25] and seemed to spread despite eradication attempts, with recent detections of two new invaded countries, Spain and Luxembourg [24]. The mosquito also spread mostly in North America presumably by several independent introductions [25–27].

*Ae*. *j*. *japonicus* has been laboratory confirmed as vector of West Nile virus [28], another virus belongs to JEV serocomplex group. *Ae*. *j*. *japonicus* could potentially be the natural vector of West Nile virus in Europe, where outbreaks have been reported in areas invaded by the mosquito [25,29]. This adds value to the vector competence assessment of this species.

Furthermore, the presence of JEV in non-endemic areas such as Europe [30,31] and Africa [32] has emphasized the importance of elucidating the migration route and determining the potential for disease spread by an invasive species. Thus, assessing the competency of *Ae*. *j*. *japonicus* as a JEV vector, particularly from a native population in Japan, is crucial in determining their disease transmission potential.

In this study, we assessed the competence of *Ae*. *j*. *japonicus* mosquitoes as a JEV vector relative to *Cx*. *tritaeniorhynchus* through exposure to three JEV genotypes (GI and GIII as the current and past dominant genotypes, respectively; and GV which has a restricted range and has never been detected in Japan), and we determined infection, dissemination, and transmission rates by either real-time quantitative polymerase chain reaction (RT-qPCR) or focus forming assay (FFA).

## Methods

### Mosquito colonies and next-generation sequencing screening

Laboratory colonies of *Ae*. *j*. *japonicus* and *Cx*. *tritaeniorhynchus* were used for the infection experiments. The *Ae*. *j*. *japonicus* and *Cx*. *tritaeniorhynchus* colonies were originated in Narita, Chiba Prefecture, Japan [33], and Yasugi, Shimane Prefecture, Japan, in 2004 and 2018, respectively. The *Ae*. *j*. *japonicus* colony was maintained at 25°C, 55%–60% relative humidity with a 16 h:8 h light:dark cycle. The *Cx*. *tritaeniorhynchus* colony was maintained at 28°C, 55%–60% relative humidity with an 11 h:13 h light:dark cycle. Both colonies were given continuous access to 3% sucrose solution.

To address any effect from insect-specific viruses (ISV), 25 female mosquitoes from each colony were subjected to viral metagenomic screening and analysis by next-generation sequencing (NGS) as previously described by Faizah et al. [34].

After viral exposure, the ISV infection status was determined by reverse-transcriptase polymerase chain reaction (RT-PCR). About 20–24 mosquitoes were processed for RNA extraction using NucleoSpin RNA (Takara Bio, Shiga, Japan) according to the manufacturer’s protocol. Body parts were homogenized in RA1 Buffer containing 1% mercaptoethanol using the TissueLyser II (QIAGEN, Venlo, Netherlands) at 25 frequency 1/s for 30 s and centrifuged at 12,000 rpm for 3 min. Homogenate was filtered, treated with DNase, and RNA eluted with 20 μl of RNase-free dH_2_O. Samples were subsequently subjected to RT-PCR by PrimeScript One-Step RT-PCR kit version 2 (Takara Bio) according to previously described RT-PCR protocol [34]. Primers used were gene specific primer sets provided in S1 Table [34,35,36] and electrophoresis was employed for the visualization.

### Cell lines

*Ae*. *albopictus* C6/36 cells (European Collection of Authenticated Cell Culture) were maintained in minimum essential medium (MEM) containing 10% heat-inactivated fetal bovine serum (FBS) and 2% non-essential amino acids at 28°C with 5% CO_2_. C6/36 cells were used to propagate virus stock prior to mosquito infection experiments. African green monkey kidney Vero cells (Japanese Collection of Research Bioresources Cell Bank) were maintained in MEM containing 10% FBS at 37°C with 5% CO_2_. Vero cells were generally used in this study for virus quantification by FFA.

### Virus strains

Details of the three different JEV genotypes (GI, GIII, and GV) used in this study are provided in S2 Table [34,37,38]. GIII and GV were gifts from Dr. Shigeru Tajima, Department of Virology, National Institute of Infectious Diseases, Japan. All strains were propagated in C6/36 cells at 28°C with 5% CO_2_ and were harvested 4–5 days post inoculation for the infection experiments.

### Infection experiments

Female mosquitoes 9–12 days old were deprived of sugar water overnight and subsequently fed an infectious blood meal through a membrane made of pig intestine using the Hemotek feeder (Hemotek Ltd, Blackburn, UK). Each infectious blood meal contained defibrinated rabbit blood (Nippon Bio-Supp. Center, Tokyo, Japan) and virus (infected cell culture supernatant) in the ratio 1:1, supplemented with 3 mM ATP serving as a phagostimulant. Each colony was fed 3 ml of infectious blood for 1 h in a dark room (for *Cx*. *tritaeniorhynchus*) or in a lighted room (for *Ae*. *j*. *japonicus*). Virus titer in blood (log10 FFU/ml) was 8.9, 8.6, and 7.1 in JEV GI, GIII, and GV infection experiment, respectively (S2 Table). The mosquitoes were then CO_2_-anesthetized and fully-fed mosquitoes were randomly divided into two cages and maintained for 7 or 14 days in a designated mosquito incubator (27°C, 3% sucrose solution provided, humidity manually checked, 16 h:8 h light:dark cycle).

### Mosquito body parts harvest and salivation

On days 7 and 14 post viral exposure, all surviving mosquitoes were cold-anesthetized for harvesting of body parts. The wings and legs were initially dissected for immobilization. Mosquito saliva was collected by inserting the proboscis into a 10-μl micropipette tip (Thermo Fisher Scientific, Waltham, MA) containing 5 μl FBS. The mosquitoes were allowed to salivate into the FBS under insectary conditions for 1 h. After salivation, the mosquitoes were confirmed alive by their response to physical stimulus. The saliva-containing FBS was then mixed with 95 μl of MEM containing 2% FBS, 2% fungizone, and 2% penicillin–streptomycin.

After saliva collection, the mosquito body parts were divided into two collection groups: abdomen–thorax and head–wings–legs. The abdomen–thorax group was used for infection rate (IR) determination, while the head–wings–legs group was used for dissemination rate (DR) determination. The body parts and saliva of each individual were stored in 1.5-ml safe-lock tubes (Eppendorf, Hamburg, Germany) at −80°C for further experiments. In this study, IR is defined as the proportion of infected individuals among the virus-tested mosquitoes; DR is the proportion of infected individuals having viral dissemination to body parts (head–wings–legs; and transmission rate (TR) is the proportion of infected individuals with virus in their saliva. Additionally, the DR and TR for each colony was determined using the total number of tested mosquitoes.

### Infection and dissemination rate determination

Viral RNA was extracted from abdomen-thorax and head-wings-legs using NucleoSpin RNA (Takara Bio) following the above-mentioned protocol. RNA was stored at −80°C until used. Viral RNA titers were subsequently determined by RT-qPCR. Standards for RT-qPCR were constructed and confirmed by ethidium bromide electrophoresis. The RT-qPCR mixture contained Taq-Man Fast Virus 1-Step Master Mix (Thermo Fisher Scientific), JEV primers (forward and reverse), 10.1 μM probe, and RNA sample. Up to 1 μl was taken from a total volume of 10 μl for quantification by PikoReal Real-Time PCR System (Thermo Fisher Scientific) under the following conditions: 1 cycle at 50°C for 5 min and 95°C for 20 s; 35 cycles at 95°C for 3 s, and 60°C for 30 s. The results were analyzed by PikoReal software version 2.2 (Thermo Fisher Scientific). Ct values above 30 were considered negative. Details of the primers and probes used in the study are provided in S1 Table.

### Confirmation of JEV in saliva

For selected samples including those positive for JEV regarding IR and DR, 60 μl of saliva in MEM (containing FBS, fungicide, and antibiotics) was used to infect Vero cells in a 24-well plate. After inoculation the cells were incubated at 37°C for 1 h before subsequently adding 440 μl medium. Supernatant was taken at 0, 3, 4, or 5 days post infection (dpi) depending on the severity of the cytopathic effect (CPE). The increase in viral RNA over time was used to identify samples containing infectious virus. In some samples, CPE was exhibited as early as 3 dpi, denoting a high viral titer which was confirmed by FFA of the initial inoculum.

A rapid viral RNA screening method was developed following the methods of Morita et al. [39] with modifications. Detergent mixture containing 1% nonidet P-40 and 5% RNaseOUT recombinant ribonuclease inhibitor (10 IU, Thermo Fisher Scientific) in phosphate buffered saline (PBS) solution was added to the cell culture supernatant. The ratio of harvested supernatant:detergent mixture was 1:1 (2.5 μl each). After incubation at room temperature for 1 min, a small amount (0.5 μl) was added to the RT-qPCR mixture containing 3 μl dH_2_O, 5 μl 2× buffer, 0.5 μl each of forward and reverse primer, and 0.5 μl enzyme mixture [PrimeScript One-Step RT-PCR kit version 2 (Takara Bio, Shiga, Japan)]. Primers used in the RT-PCR were a JEV universal primer set (forward: 5′-GCYGARCAGAAYCAATGGAGC-3′; and reverse: 5′-GCTCCWAGCCACATGAACCA-3′) with a resultant band length of 202 bp. A total volume of 10 μl was amplified under the following RT-qPCR conditions: 1 cycle at 50°C for 30 s, 94°C for 2 min; 35 cycles at 94°C for 15 s, 53°C for 15 s, 72°C for 30 s. The PCR results were then visualized by electrophoresis. All samples were run with positive and negative controls.

### Focus forming assay

Vero cells were used for viral titration in the FFA. The cells were seeded in 12-well plates at a concentration of 2 × 10^5^ cells/well. A 100-fold dilution of saliva samples was prepared in MEM and 100 μl added to the wells. After incubation for 1 h at 37°C the inoculum was removed, and 1 ml of MEM supplemented with methylcellulose and 2% FBS was added to each well and incubated at 37°C for 3 days. Without removing the methylcellulose overlay, the cells were fixed by adding 2.6% paraformaldehyde to each well followed by incubation at room temperature (RT) for 20 min, followed by a PBS wash before adding 0.1% Triton-X100 for an extra 20-min incubation at RT. After the fixation step, cells were washed with PBS and incubated for 1 h at 37°C with mouse monoclonal anti-flavivirus group antigen antibody harvested from hybridoma cells (D1-4G2-4-15, ATCC HB-112), followed by 30 min incubation at RT with the secondary antibody (Dako Envision + System HRP labelled polymer, Agilent Technologies, Santa Clara, CA) on a slow-speed laboratory shaker. Finally, the substrate (Dab+Chromosome substrate kit, Agilent Technologies) was added and the resulting viral titer was determined.

### Statistical analysis

Two-tailed Fisher’s exact test using 2x2 contingency table corrected with Bonferroni’s method was used for calculating the difference in proportions of IR, DR, and TR. Nonparametric Kruskal-Wallis test corrected with Bonferroni’s method was also performed for statistical analysis of viral titers. For ease of viewing in the scatter/dot plots, JEV RNA copy levels were log-transformed. GraphPad Prism software version 8 and Microsoft Excel 2013 were used for statistical analysis and value transformation.

## Results

### Metagenomic analysis of mosquito colonies and ISV infection status

Based on metagenomics analysis, the *Cx*. *tritaeniorhynchus* colony was confirmed to be persistently infected by a new strain of Cx. vishnui subgroup totivirus (CvsTV strain Shimane, accession number LC567881), and the *Ae*. *j*. *japonicus* colony was confirmed to be persistently infected by a new strain of Aedes japonicus narnavirus 1 (AejapNV1 strain Narita, accession number LC567882).

CvsTV strain Shimane had 99.7% identity and 100% query cover by blastn compared to the previously described CvsTV strain (accession no. LC514295). The genome obtained was 7795 nt in length and had two ORFs encoding putative capsid and RNA-dependent RNA polymerase (RdRp). AejapNV1 strain Narita had 97.9% identity and 91% query cover by blastn compared to the original AejapNV1 strain (accession no. MK984721). The genome obtained was 3144 nt in length with 1026 amino acids (aa) in the RdRp and 1030 aa in a hypothetical protein (S1 Fig).

Results from ISV infection status showed that the *Cx*. *tritaeniorhynchus* mosquito colony harbored CvsTV strain Shimane, while the *Ae*. *j*. *japonicus* colony harbored AejapNV1 strain Narita (S3 Table). There was no observable effect of ISV on JEV infection.

### JEV infection (IR) and dissemination rates (DR) in the two mosquito species

#### Cx. tritaeniorhynchus

A total of 274 out of 367 (75%) *Cx*. *tritaeniorhynchus* mosquitoes exposed to JEV were successfully screened for JEV infection. Colonies exposed to JEV GV accounted for over two thirds of the mosquitoes that died upon exposure to JEV (S2 Fig).

From the 274 tested, as much as 261 mosquitoes (95%) were infected. The colony generally displayed high IR, ranging from 77–100% (Table 1). The JEV GIII and GV, both had an IR of 100% and 98% at 7 and 14 dpi, respectively, whereas the IR for GI was lower but with an increase between the two time points of collection; 77% at 7 dpi and 92% at 14 dpi (Table 1).

**Table 1:** Comparisons of the infection (IR), dissemination (DR), and transmission rates (TR) of *Cx*. *tritaeniorhynchus* and *Ae*. *j*. *japonicus* infected with three different genotypes of JEV.

	7 dpi			14 dpi			Combined	Combined (per total infected)		Combined (per total tested)	
	IR	DR	TR	IR	DR	TR	IR	DR	TR	DR	TR
*Cx*. *tritaeniorhynchus*											
**JEV GI**	27/35 (77%)a	26/35 (74%)a	23/27 (85%)a	37/40 (92%)a	35/40 (88%)a	34/37 (92%)a	64/75 (85%)a	61/64 (95%)a	57/64 (89%)a	61/75 (81%)a	57/75 (76%)a
**JEV GIII**	49/49 (100%)b	46/49 (94%)ab	38/49 (78%)a	48/49 (98%)a	48/49 (98%)a	44/48 (92%)a	97/98 (99%)b	94/97 (97%)a	82/97 (85%)a	94/98 (96%)b	82/98 (84%)b
**JEV GV**	51/51 (100%)b	49/51 (96%)b	45/51 (88%)a	49/50 (98%)a	48/50 (96%)a	45/49 (92%)a	100/101 (99%)b	97/100 (97%)a	90/100 (90%)a	97/101 (96%)b	90/101 (89%)ab
*Ae*. *j*. *japonicus*											
**JEV GI**	3/25 (12%)ab	3/25 (12%)ab	2/3 (67%)a	0/23a	0/23a	N/A	3/48 (6%)ab	3/3 (100%)a	2/3 (67%)a	3/48 (6%)ab	2/48 (4%)ab
**JEV GIII**	7/32 (22%)a	7/32 (22%)a	5/7 (71%)a	5/32 (16%)a	5/32 (16%)a	5/5 (100%)a	12/64 (19%)a	12/12 (100%)a	10/12 (83%)a	12/64 (19%)a	10/64 (16%)a
**JEV GV**	0/36b	0/36b	N/A	1/29 (3%)a	1/29 (3%)a	1/1 (100%)a	1/65 (2%)b	1/1 (100%)a	1/1 (100%)a	1/65 (2%)b	1/65 (2%)b

Infection, dissemination and transmission rates between the three genotypes in a collection time point were compared within species (*Cx*. *tritaeniorhynchus* or *Ae*. *j*. *japonicus*). The data has been separated into; *Cx*. *tritaeniorhynchus* and *Ae*. *japonicus* exposure, at 7 and 14 dpi each, and combined percentages, both per total of infected individuals and total tested. Fisher’s exact test corrected with Bonferroni’s method was applied. Statistical significance between genotypes are represented by letters in red (a, b, or ab). Genotypes that share the same red letter in a column are not significantly different. N/A, not applicable.

The mosquito also showed variations in the total numbers of infected individuals between the three genotypes, lowest in the GI group (Table 1). In particular, at 7 dpi the IR was significantly lower in GI versus GIII and GV (*p* < 0.0001) with only 27 out of 35 mosquitoes infected. By 14 dpi no significant differences existed between the genotypes.

The DR corresponded well with the IR, with a slight difference in the number of individuals having dissemination in secondary body parts. However, in general, DR results reflected those for IR, with significant difference displayed at 7 dpi with GI being significantly lower than GV, but not GIII, and no significant difference observed at 14 dpi (Table 1).

**Fig 3:**
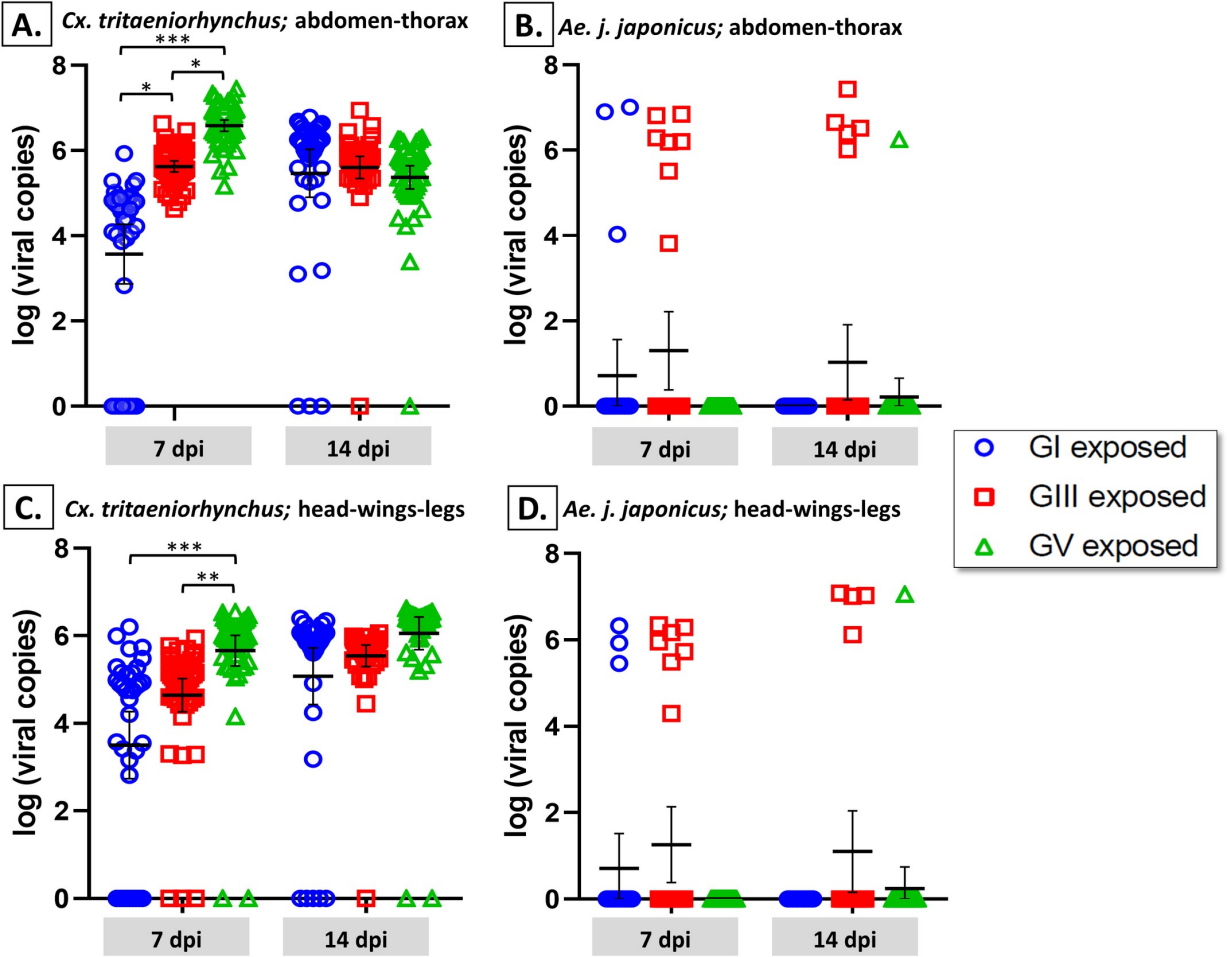
**Differences in viral load of *Cx*. *tritaeniorhynchus* (A and C) and *Ae*. *j*. *japonicus* (B and D) infected with three different genotypes of JEV (GI, GIII and GV).** Mosquitoes were fed a virus–blood mixture containing either GI, GIII or GV. At 7 and 14 days post infection (dpi), the abdomen–thorax (A and B) and head–wings–legs (C and D) were harvested from individual mosquitoes and the viral load titrated using RT-qPCR. The bars represent the mean with 95% confidence interval (CI). Multiple comparisons of proportions using Kruskal Wallis corrected with Bonferroni’s method was applied to determine significant differences between JEV genotypes at each time point. Only statistically significant differences are shown by the asterisks; ***, *p* < 0.0001; **, *p* < 0.001; *, *p* < 0.01.

Viral titer results showed significant differences between genotypes at the two time points, particularly at 7 dpi (Fig 3A and 3C). Viral load in the abdomen–thorax at 7 dpi showed significant differences between all genotypes (*p* < 0.01), while at 14 dpi, no significant differences displayed (Fig 3A). In the secondary body parts (head–wings–legs), similar results were exhibited with significant differences at 7 dpi with GV being significantly higher than GI and GIII (*p* < 0.001) and no significant differences displayed at 14 dpi (Fig 3C).

Between the two time points, there were differences observed in the viral load peaks. Thus, to make a comparison between the two time points, mean viral titers of the infected mosquitoes were plotted, indicating that each genotype displayed a different trendline from 7 to 14 dpi (S3 Fig).

#### Ae. j. japonicus

A total of 177 out of 244 (73%) *Ae*. *j*. *japonicus* mosquitoes exposed to JEV were tested for JEV infection. Similarly, colonies exposed to JEV GV accounted for over two thirds of the mosquitoes that died upon exposure to JEV (S2 Fig).

From those 177 tested, only 16 mosquitoes (9%) were infected. This colony showed low IR, ranging from 0%–22% (Table 1) JEV GIII had the highest IR, with 22% and 16% at 7 and 14 dpi, respectively. IR in GI and GV was ≤12% at all time points (Table 1). To examine any difference between genotypes, the number of infected mosquitoes, regardless of time points, was compared by multiple comparisons of proportions using multiple comparisons by Fisher’s exact test corrected with Bonferroni’s method, and a significant difference was found between GIII and GV at 7 dpi (*p* < 0.01), with 7 out of 32 mosquitoes infected in GIII. By 14 dpi, no significant differences were found between the genotypes (Table 1).

Viral load results for both body parts, the abdomen–thorax and the head–wings–legs, showed that there were no significant differences observed between genotypes at two collection time points (Fig 3B and 3D).

To make a comparison between the two time points, mean viral titers of the JEV GIII-infected mosquitoes were plotted, showing that there was a slight increase from 7 to 14 dpi (S4 Fig). Low IR precluded similar comparisons for the GI and GV groups.

### JEV transmission rates (TR) in the *Cx*. *tritaeniorhynchus* and *Ae*. *j*. *japonicus* species

#### Cx. tritaeniorhynchus

TR was defined as the number of individuals with virus in their saliva per total infected mosquitoes, and results showed that there were no significant differences between the virus genotypes (Table 1). Between 85% and 90% of the total of infected mosquitoes, or 76 and 89% of the tested mosquitoes, had viruses in their saliva.

The average viral titer produced during the one-time salivation in C*x*. *tritaeniorhynchus* was 1.62E+05 ffu/ml (Table 2), with the highest viral titers reached being 1.80E+06, 5.93E+06, and 9.00E+04 ffu/ml in GI at 14 dpi, GIII at 14 dpi, and GV at 7 dpi, respectively.

**Table 2:** Mean viral titers of three JEV genotypes in infected *Cx*. *tritaeniorhynchus* and *Ae*. *j*. *japonicus* mosquitoes.

Source	dpi	GI	GIII	GV	Mean	log_10_
***Cx*. *tritaeniorhynchus***						
Abdomen–thorax	7	9.09E+04	6.95E+05	6.24E+06	1.70E+06	6.23
	14	1.75E+06	8.53E+05	5.58E+05		
Head–wings–legs	7	2.08E+05	1.66E+05	1.13E+06	1.26E+06	6.10
	14	9.28E+05	5.38E+05	4.61E+06		
Saliva*	7	7.31E+03	2.18E+04	3.14E+04	1.62E+05	5.21
	14	1.67E+05	7.10E+05	3.55E+04		
***Ae*. *j*. *japonicus***						
Abdomen–thorax	7	6.01E+06	3.13E+06	N/A	4.63E+06	6.67
	14	N/A	7.61E+06	1.76E+06		
Head–wings–legs	7	1.09E+06	1.06E+06	N/A	1.07E+07	7.03
	14	N/A	2.90E+07	1.16E+07		
Saliva*	7	N/A	3.37E+04	N/A	4.68E+04	4.67
	14	N/A	6.00E+04	N/A		

*Mean viral titers in saliva were calculated only using quantifiable results from FFA (>10^2^ ffu/ml)

#### Ae. j. japonicus

There were no significant differences in TR for all genotypes (Table 1). In combined results regardless of collection time points, the percentages were 67% to 100% of the total of infected mosquitoes, or only 2 to 16% of tested mosquitoes (Table 1). The highest viral titer was 6.00E+04 ffu/ml in GIII at 14 dpi (Table 2).

### Comparisons of IR, DR, and TR between the *Cx*. *tritaeniorhynchus* and *Ae*. *j*. *japonicus* species

**Fig 4:**
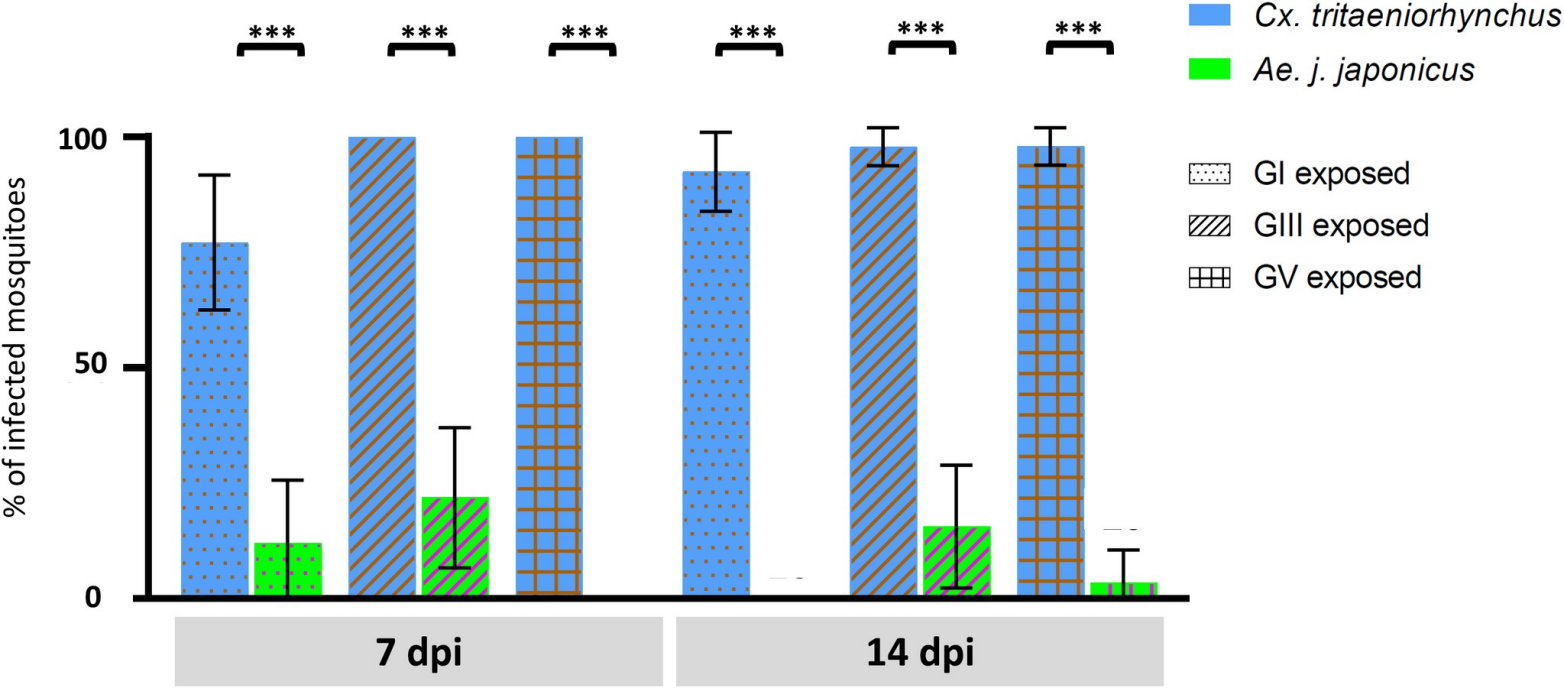
Comparisons of proportion of infected adults (%) between *Cx*. *tritaeniorhynchus* and *Ae*. *j*. *japonicus* tested to three different genotypes of JEV (GI, GIII, and GV). *Cx*. *tritaeniorhynchus* (blue bars) or *Ae*. *j*. *japonicus* (green bars) mosquitoes were tested to JEV GI, GIII or GV strains. At 7 and 14 days post infection (dpi), the abdomen–thorax were harvested from individuals and the viral load titrated using RT-qPCR. The bars represent the proportion of individuals infected as a percentage and error bars represent the 95% confidence interval. Fisher’s exact test (two-tailed) corrected with Bonferroni’s method was applied to determine significant differences between the two mosquito species at the same time point and genotype. *, *p* < 0.0001.

There were large and significant differences in IR between the two species of mosquito at both time points and for all three genotypes (*p* < 0.0001) (Fig 4).

After viral quantification, mean viral titers were obtained and the results (log_10_ transformed) are shown in Table 2. Viral loads in abdomen–thorax and secondary body parts (head–wings–legs) in *Cx*. *tritaeniorhynchus* were 6.23 and 6.10, respectively, while those in *Ae*. *j*. *japonicus* were 6.67 and 7.03, respectively. The mean viral titers in saliva (log_10_) were 5.21 and 4.67 in *Cx*. *tritaeniorhynchus* and *Ae*. *j*. *japonicus*, respectively.

Two-tailed Fisher’s exact test was used to compare total TR between the two species of mosquito at all time points and no significant difference was found (Table 1). In total, 229 out of 274 tested ***C****x*. *tritaeniorhynchus* mosquitoes (84%) had virus in the saliva, while in *Ae*. *j*. *japonicus* mosquitoes, the figure was 13 out of 177 infected (7%).

## Discussion

*Cx*. *tritaeniorhynchus* is known as the primary vector in most countries where JE is endemic, and studies have shown that all five defined genotypes of JEV can be successfully isolated from field-collected *Cx*. *tritaeniorhynchus* [9,40,41]. However, confirmation from laboratory experiments has so far been limited due to difficulties in *Cx*. *tritaeniorhynchus* laboratory colony establishment and maintenance. In this study, having successfully established a laboratory colony, we exposed *Cx*. *tritaeniorhynchus* to three of the JEV genotypes (GI, GIII, and GV) and compared infection rates.

*Ae*. *j*. *japonicus* has long been suspected as a possible secondary JEV vector, and the successful transmission of a GIII strain (JANAr-5681) in this species has been experimentally tested [42]. However, that study was limited, and no data are currently available regarding infection with genotypes other than GIII. Here, under the same experimental conditions, we compared the competency of both *Cx*. *tritaeniorhynchus* and *Ae*. *j*. *japonicus* as vectors of three JEV genotypes.

Results showed *Cx*. *tritaeniorhynchus* to be highly susceptible, as almost all individuals tested were infected by the virus, had dissemination throughout their body parts, and successfully released the viral particles through their saliva, thus confirming their competency as a JEV vector. In contrast, *Ae*. *j*. *japonicus* did not show the same level of susceptibility. However, a small proportion did become infected and did release infectious virus particles in their saliva.

The ability of *Ae*. *j*. *japonicus* to invade new geographical regions, in addition to their high adaptability in new environments, may pose a public health risk, particularly in relation to pathogen introduction over wider areas. In this study, we have demonstrated that *Ae*. *j*. *japonicus* (strain Narita) can transmit JEV, albeit not as efficiently as the primary vector, *Cx*. *tritaeniorhynchus*.

This study demonstrated the robust vector competency of *Cx*. *tritaeniorhynchus* in transmitting three JE genotypes. This may reflect the mosquito strain, as it has been reported that two out of three strains of *Cx*. *tritaeniorhynchus* originating from Japan were highly efficient transmitters compared to other strains from different Asian countries [43]. Due to its high susceptibility to JEV the *Cx*. *tritaeniorhynchus* strain used in this study may be ideal as a positive control for future JEV mosquito infection studies.

The length of extrinsic incubation seemed to vary between genotypes, even in the JE primary vector, with GIII and GV exhibiting a somewhat longer period compared to GI. Plots of the mean viral titers of infected mosquitoes at 7 and 14 dpi showed three trends: increasing (GI), level (GIII), or slightly decreasing (GV). Variation in extrinsic incubation period (EIP; i.e. the interval between ingestion of a virus by a mosquito and the mosquito's ability to transmit the pathogen to a susceptible vertebrate host) was suspected as the cause of the difference among genotypes, with GI being slightly later in reaching the peak compared to GIII and GV based on mean viral titers in infected mosquitoes. Differential infectivity and EIP among JEV genotypes have been demonstrated by *Cx*. *quinquefasciatus* in North America [44,45], though no differences were seen in *Cx*. *pipiens* in China [46].

Worldwide, there are five JEV genotypes, with GI and GIII being the dominant lineages, and GII, GIV, and GV found in restricted areas. The mechanism behind genotype displacement from GIII to GI in East Asia in the 1990s is still unknown. The GIII strain used in this study displayed a longer EIP while maintaining its viral titer level in *Cx*. *tritaeniorhynchus* (strain Shimane) inferring better compatibility with the mosquito host than GI. JEV GIII strain JaGAr01, which is presumed to be indigenous to Japan, showed higher infectivity at 7 dpi compared to GI and maintained the established titer to the other collection time point (14 dpi). This may infer that genotype displacement has no connection to the vector competence of *Cx*. *tritaeniorhynchus*.

Among the other JEV genotypes used in this study, GV showed a similar robust competency, on par with GIII. Furthermore, the mean viral titers at 7 dpi were slightly higher than GIII at all time points. This finding suggests that *Cx*. *tritaeniorhynchus* is as susceptible to and as competent at transmitting GV as it is for GIII. JEV GV was isolated from *Cx*. *tritaeniorhynchus* mosquitoes in China in 2009 [9] and from human specimens in South Korea in 2015 [11]. It was also detected in *Cx*. *bitaeniorhynchus* collected in South Korea in 2010 [10], whereas in Japan, GV has never been detected. National mosquito surveillance and arboviral disease surveillance must, therefore, be conducted periodically in order to detect and potentially prevent JEV GV introduction or outbreaks.Under the same experimental conditions as for *Cx*. *tritaeniorhynchus*, *Ae*. *j*. *japonicus* (strain Narita) was found to be susceptible to JEV infection, albeit with a significantly lower IR. Although the numbers infected were low, virus progression within the mosquito was efficient, leading to a high TR per total of infected mosquitoes. This finding was confirmed by the correlation between IR and DR, followed by a high TR, with 100% transmissibility of JEV GIII and GV at 14 dpi. This may highlight the importance of the midgut infection barrier as a vector competence indicator in *Ae*. *j*. *japonicus*. The efficient infectivity displayed by *Ae*. *j*. *japonicus* was evidenced not only by consistent numbers of positive samples but also by the established viral titer in infected individuals, comparable to those observed in *Cx*. *tritaeniorhynchus*.

When comparisons between genotypes showed no significant differences, *Ae*. *j*. *japonicus* had better compatibility with JEV GIII in terms of percentage of infected individual. It is difficult to assess the length of the EIP in genotypes other than GIII in *Ae*. *j*. *japonicus* due to the low number of positive samples. Nevertheless, our study has demonstrated that *Ae*. *j*. *japonicus* is more susceptible to JEV GIII than to GI or GV. With a prolonged EIP, JEV GIII displayed higher IR and DR compared to the other genotypes. Importantly, JEV GIII is the only genotype that was transmitted through *Ae*. *j*. *japonicus* saliva at very high titer, reaching 6.00E+04 ffu/ml.

Our results are in line with those of the only other report on *Ae*. *j*. *japonicus* vector competency to JEV GIII [42], which reported *Ae*. *j*. *japonicus* transmissibility of 33% following exposure to viremic chicks, and 75% after exposure to blood-soaked cotton. *Ae*. *j*. *japonicus* certainly has the ability to become a JEV vector in places where the primary vector is scarce or absent. Cases of JEV have been recorded in many places outside its endemic area, including Italy [30,31] and Angola [32]. The JEV entry route in these two countries is still unknown but the risk from an invasive species stepping in as a competent vector has been demonstrated.

Not only does this study offer a possible explanation for the JEV outbreaks and infrequent detection in primary vector-free areas in Hokkaido, Japan, but it also highlights the risk of JEV spreading outside its endemic areas. The risk of *Ae*. *j*. *japonicus* carrying JEV into wider areas raises a public health concern, especially if the mosquito is competent enough to transmit to the next host. In the worst possible situation, a single mosquito could carry the virus, adapt to the new environment, and transmit it to an amplifier host, such as wild boar or swine. Subsequently, local mosquitoes could play the role of maintaining the JEV cycle. A study has demonstrated that European mosquito species including *Ae*. *albopictus* and *Cx*. *pipiens* are also competent vectors for JEV [47], thus, exposure to the virus may result in an outbreak.

Another point to note is the detection of the same virus species persistently infecting the *Ae*. *j*. *japonicus* mosquito (AejapNV1), and recently discovered in an established *Ae*. *j*. *japonicus* population in the Netherlands [48]. The genome obtained in this study was 3144 nt in length with 1026 aa in the RdRp and 1030 aa in a hypothetical protein, longer than a previously reported sequence. The high blastn identity result (98%) with AejapNV1 (accession no. MK984721) implies that the virus is an ISV infecting *Ae*. *j*. *japonicus* mosquitoes. There was no observable effect displayed by the persistently infecting viruses to the current infection studies using *Cx*. *tritaeniorhynchus* and *Ae*. *j*. *japonicus*. In this study we also noticed the possible effect of JEV genotype exposure on mosquito survival. Percentage death rate of both mosquito species was significantly higher after exposure to JEV GV compared to other genotypes. A study by Gaye et al. [49] comprehensively revealed disparities in *Aedes* mosquito survival on exposure to different DENV serotypes. While more studies will be needed to explain this observation, this may provide insight into the reason for the absence of JEV GV in *Cx*. *tritaeniorhynchus* in Japan or the limited detection in other endemic countries.

Occurrences of JEV in Europe and Africa may indicate that the two continents could become new endemic areas [8], and this study may have emphasized the notion. Although, the caveat is that vector competence is only one of several vectorial capacity components that determine a species ability to act as a competent vector in acquiring and later transmitting a virus [50]. For example, variations in temperature have been found to affect vector competence, both under laboratory conditions [51] and in nature [52]. Mosquito density and longevity have also been strongly linked to vectorial capacity, with one study describing the ability of an incompetent vector in causing a disease outbreak due to their high population density in the area [53].

Therefore, although cautious interpretation may need to be applied when using mosquito colonies adapted to laboratory condition instead of wild population due to the possible impact of reduced genetic variability on susceptibility [54,55,56], a study has inferred that an established laboratory colony may be a representative model [45]. That being the case, future studies using semi-field colonies and/or wild populations will still be essential in completely understanding JEV vector competence, and finally elucidating the likelihood of an endemic transmission cycle establishment through an invasive species.

Nonetheless, this study has confirmed the robust competence of *Cx*. *tritaeniorhynchus* in transmitting three JEV genotypes. To the best of our knowledge, this is the first infection study of JEV GV in *Cx*. *tritaeniorhynchus*. Further, this study has also demonstrated the ability of *Ae*. *j*. *japonicus* in transmitting three JEV genotypes. To our knowledge, this is the first infection study of JEV genotypes other than GIII in *Ae*. *j*. *japonicus* originating from a JE-endemic country.

## Supporting information

S1 Fig Genome structure of of Aedes japonicus Narnavirus 1 (AejapNV1) strain Narita. Obtained AejapNV1 strain Narita genome was 3144 nt in length with 1026 amino acids (aa) in the RdRp and 1030 aa in a hypothetical protein. Two open reading frames (ORF) containing ORF 1 and ORF 2, encode an RNA-dependent RNA polymerase and hypothetical protein, respectively.
(TIF)

S2 Fig The percentages of mosquitoes that died after JEV exposure. Error bars represent the standard deviation. Two-tailed fisher’s exact test corrected with Bonferroni’s method was applied to determine significant differences between genotypes. *, *p* < 0.0001.
(TIF)

S3 Fig Variations of the means of viral titers of different JEV genotypes in 7 and 14 days post infection. Mean viral titers of the infected mosquitoes, collected in either 7 or 14 dpi, revealed different courses: increase (GI), stationary (GIII) or slight decrease (GV).
(TIF)

S4 Fig Mean viral titers of JEV GIII in infected *Ae j*. *japonicus*. (TIF)

S1 Table Primers and probes used in the study. (XLSX)

S2 Table JEV strains used in the study. (XLSX)

S3 Table ISV infection status in tested mosquitoes. (XLSX)
