# Correction: Evaluating the competence of the primary vector, *Culex tritaeniorhynchus*, and the invasive mosquito species, *Aedes japonicus japonicus*, in transmitting three Japanese encephalitis virus genotypes

**DOI:** 10.1371/journal.pntd.0011052

**Published:** 2023-01-12

**Authors:** Astri Nur Faizah, Daisuke Kobayashi, Michael Amoa-Bosompem, Yukiko Higa, Yoshio Tsuda, Kentaro Itokawa, Kozue Miura, Kazuhiro Hirayama, Kyoko Sawabe, Haruhiko Isawa

There are errors to the numbering of the references. References 37–56 are misnumbered. The correct references 37–56 are as follows:

37. Mangada MNM, Takegami T. Molecular characterization of the Japanese encephalitis virus representative immunotype strain JaGAr 01. Virus Res. 1999;59(1):101–112. pmid:10854169

38. Tajima S, Yagasaki K, Kotaki A, Tomikawa T, Nakayama E, Moi ML, et al. In vitro growth, pathogenicity and serological characteristics of the Japanese encephalitis virus genotype V Muar strain. J Gen Virol. 2015;96(9):2661–2669. pmid:26048886

39. Morita K, Tanaka M, Igarashi A. Rapid identification of dengue virus serotypes by using polymerase chain reaction. J Clin Microbiol. 1991;29(10):2107–2110. pmid:1682341

40. Rosen L. The natural history of Japanese encephalitis virus. Annu Rev Microbiol. 1986;40:395–414. pmid:2877613

41. Schuh AJ, Guzman H, Tesh RB, Barrett ADT. Genetic diversity of japanese encephalitis virus isolates obtained from the indonesian archipelago between 1974 and 1987. Vector-Borne and Zoonotic Diseases. 2013;13(7):479–488. pmid:23590316

42. Takashima I, Rosen L. Horizontal and vertical transmission of japanese encephalitis virus by aedes japonicus(Diptera: culicidae). Journal of Medical Entomology. 1989;26(5):454–458. pmid:2552120

43. Takahashi M. Differential transmission efficiency for Japanese encephalitis virus among colonized strains of Culex tritaeniorhynchus. Med Entomol Zool. 1982;33(4):325–333.

44. Huang Y-JS, Hettenbach SM, Park SL, Higgs S, Barrett ADT, Hsu W-W, et al. Differential infectivities among different japanese encephalitis virus genotypes in culex quinquefasciatus mosquitoes. Charrel R, editor. PLoS Negl Trop Dis. 2016;10(10):e0005038. pmid:27706157

45. Karna AK, Bowen RA. Experimental evaluation of the role of ecologically-relevant hosts and vectors in japanese encephalitis virus genotype displacement. Viruses. 2019;11(1):32. pmid:30621345

46. Hameed M, Liu K, Anwar MN, Wahaab A, Safdar A, Di D, et al. The emerged genotype I of Japanese encephalitis virus shows an infectivity similar to genotype III in Culex pipiens mosquitoes from China. Smith D, editor. PLoS Negl Trop Dis. 2019;13(9):e0007716. pmid:31557156

47. de Wispelaere M, Desprès P, Choumet V. European aedes albopictus and culex pipiens are competent vectors for japanese encephalitis virus. Turell MJ, editor. PLoS Negl Trop Dis. 2017;11(1):e0005294. pmid:28085881

48. Abbo SR, Visser TM, Wang H, Göertz GP, Fros JJ, Abma-Henkens MHC, et al. The invasive Asian bush mosquito Aedes japonicus found in the Netherlands can experimentally transmit Zika virus and Usutu virus. Failloux A-B, editor. PLoS Negl Trop Dis. 2020;14(4):e0008217. pmid:32282830

49. Gaye A, Wang E, Vasilakis N, Guzman H, Diallo D, Talla C, et al. Potential for sylvatic and urban Aedes mosquitoes from Senegal to transmit the new emerging dengue serotypes 1, 3 and 4 in West Africa. Dinglasan RR, editor. PLoS Negl Trop Dis. 2019;13(2):e0007043. pmid:30759080

50. Kenney JL, Brault AC. The role of environmental, virological and vector interactions in dictating biological transmission of arthropod-borne viruses by mosquitoes. In: Advances in Virus Research [Internet]. Elsevier; 2014 [cited 2020 Sep 8]. p. 39–83. Available from: https://linkinghub.elsevier.com/retrieve/pii/B9780128001721000021

51. Liu Z, Zhang Z, Lai Z, Zhou T, Jia Z, Gu J, et al. Temperature increase enhances aedes albopictus competence to transmit dengue virus. Front Microbiol. 2017;8:2337. pmid:29250045

52. Carrington LB, Armijos MV, Lambrechts L, Scott TW. Fluctuations at a low mean temperature accelerate dengue virus transmission by aedes aegypti. PLoS Negl Trop Dis. 2013;7(4):e2190. pmid:23638208

53. Miller B. R., et al. “Epidemic Yellow Fever Caused by an Incompetent Mosquito Vector.” Tropical Medicine and Parasitology: Official Organ of Deutsche Tropenmedizinische Gesellschaft and of Deutsche Gesellschaft Fur Technische Zusammenarbeit (GTZ), vol. 40, no. 4, Dec. 1989, pp. 396–399. pmid:2623418.

54. Conn JE, Lainhart W, Rios CT, Vinetz JM, Bickersmith SA, Moreno M. Changes in genetic diversity from field to laboratory during colonization of anopheles darlingi root(Diptera: culicidae). Am J Trop Med Hyg. 2015;93(5):998–1001. pmid:26283742

55. Gloria-Soria A, Soghigian J, Kellner D, Powell JR. Genetic diversity of laboratory strains and implications for research: The case of Aedes aegypti. Barker CM, editor. PLoS Negl Trop Dis. 2019;13(12):e0007930. pmid:31815934

56. Lorenz L, Tabachnick WJ, Wallis GP, Beaty BJ, Aitken THG. The effect of colonization upon aedes aegypti susceptibility to oral infection with yellow fever virus. Am J Trop Med Hyg. 1984;33(4):690–694. pmid:6476217

## References

[1] Faizah AN, Kobayashi D, Amoa-Bosompem M, Higa Y, Tsuda Y, Itokawa K, et al. (2020) Evaluating the competence of the primary vector, *Culex tritaeniorhynchus*, and the invasive mosquito species, *Aedes japonicus japonicus*, in transmitting three Japanese encephalitis virus genotypes. PLoS Negl Trop Dis 14(12): e0008986. doi: 10.1371/journal.pntd.0008986 33370301 PMC7793266

