# Supplementary figures and images for: Evaluating the competence of the primary vector, Culex tritaeniorhynchus, and the invasive mosquito species, Aedes japonicus japonicus, in transmitting three Japanese encephalitis virus genotypes
Source: PLoS Negl Trop Dis. 2020 Dec 28;14(12):e0008986. doi: 10.1371/journal.pntd.0008986 (PMC7793266; doi:10.1371/journal.pntd.0008986)

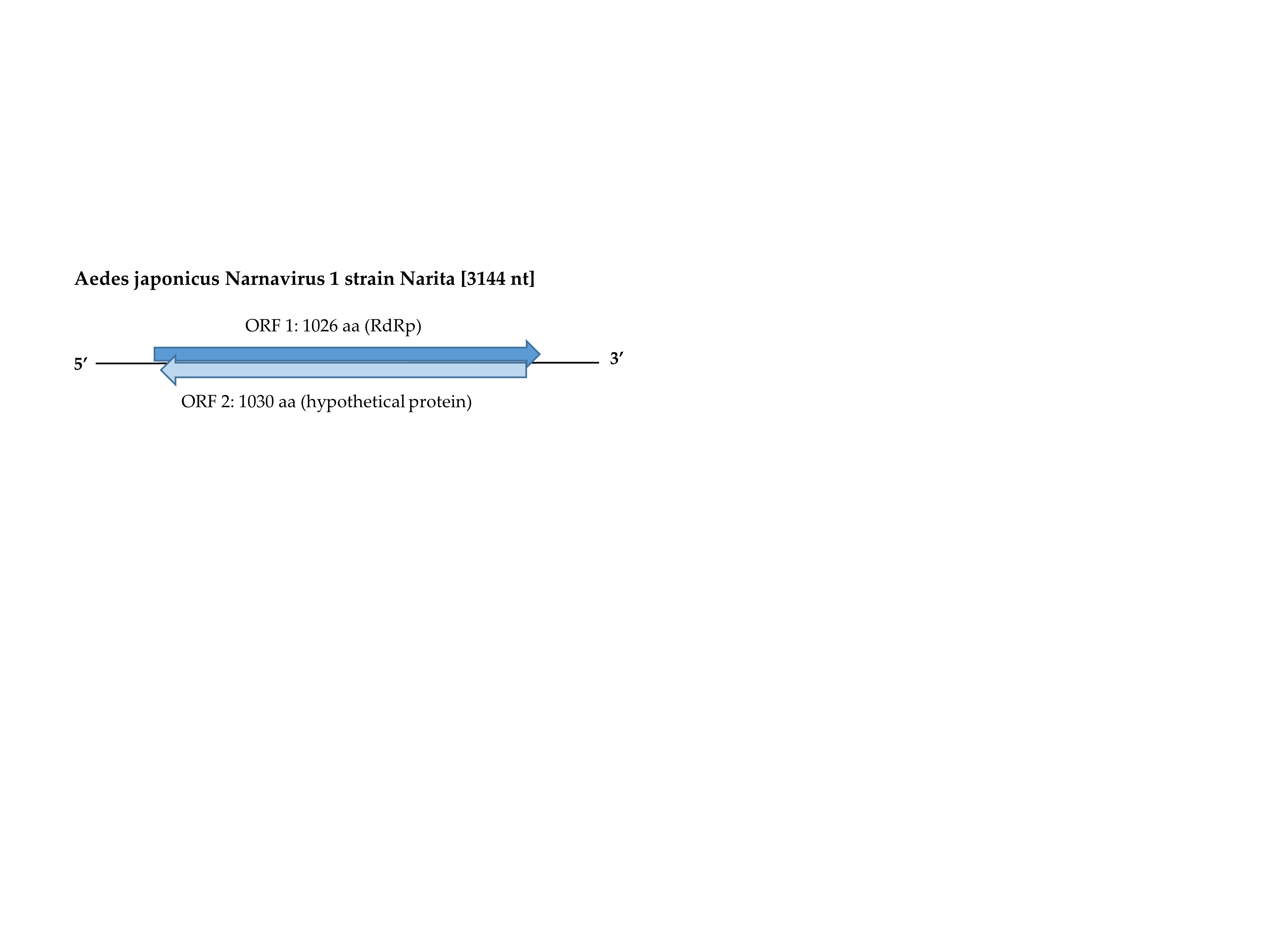

Supplement: S1 Fig — Obtained AejapNV1 strain Narita genome was 3144 nt in length with 1026 amino acids (aa) in the RdRp and 1030 aa in a hypothetical protein. Two open reading frames (ORF) containing ORF 1 and ORF 2, encode an RNA-dependent RNA polymerase and hypothetical protein, respectively. (TIF) [file pntd.0008986.s001.TIF]

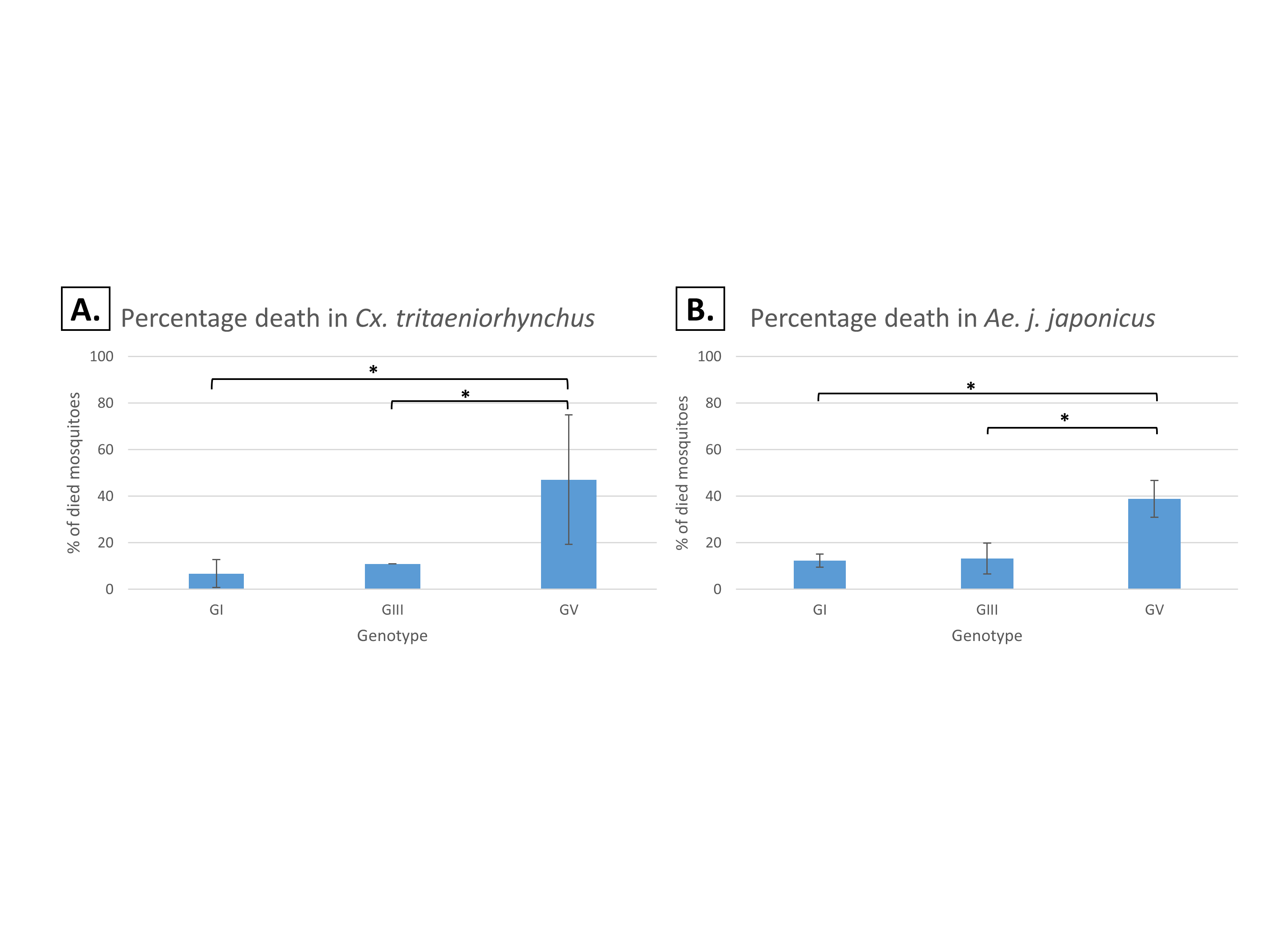

Supplement: S2 Fig — Error bars represent the standard deviation. Two-tailed fisher’s exact test corrected with Bonferroni’s method was applied to determine significant differences between genotypes. *, p < 0.0001. (TIF) [file pntd.0008986.s002.TIF]

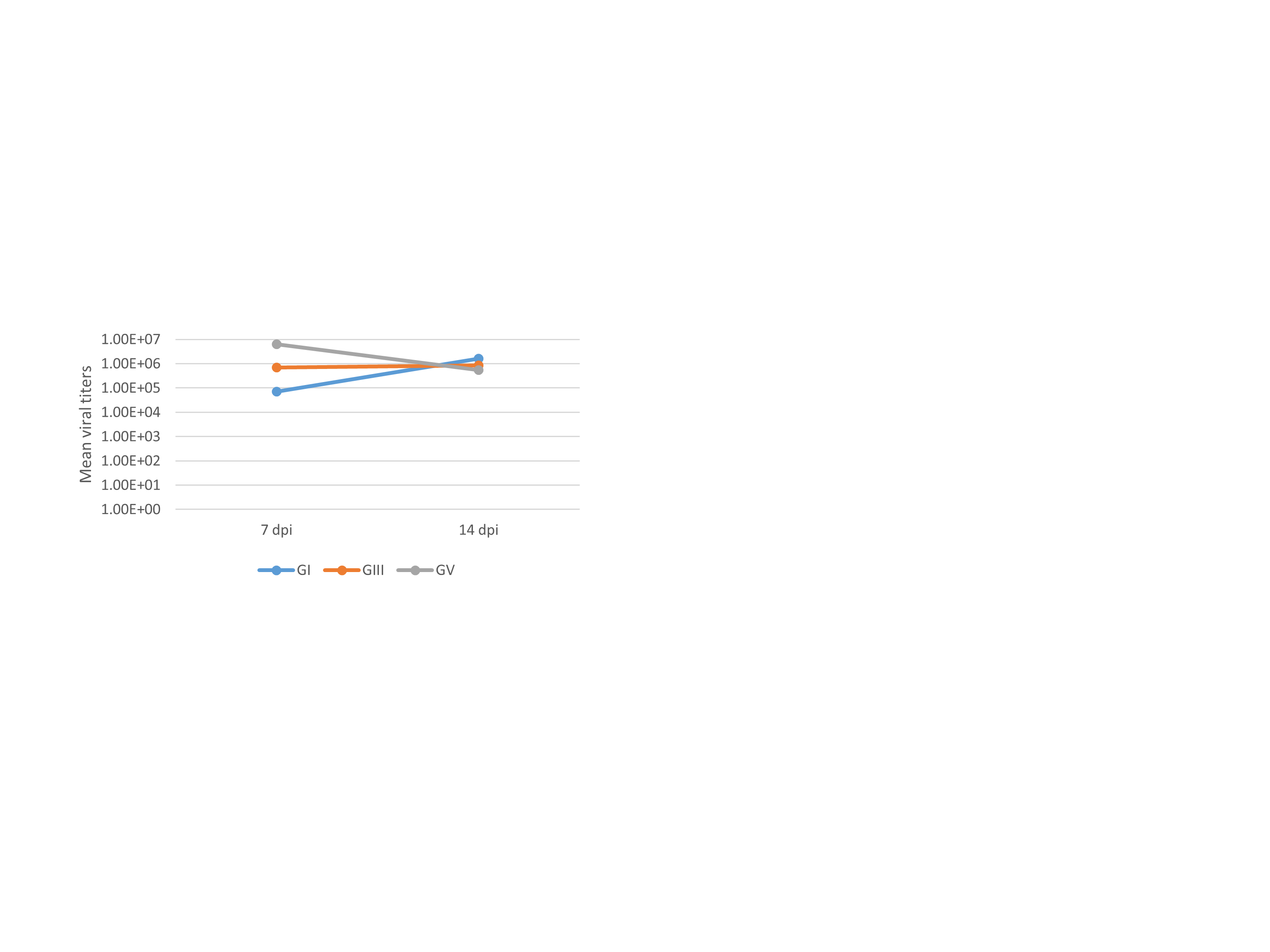

Supplement: S3 Fig — Mean viral titers of the infected mosquitoes, collected in either 7 or 14 dpi, revealed different courses: increase (GI), stationary (GIII) or slight decrease (GV). (TIF) [file pntd.0008986.s003.TIF]

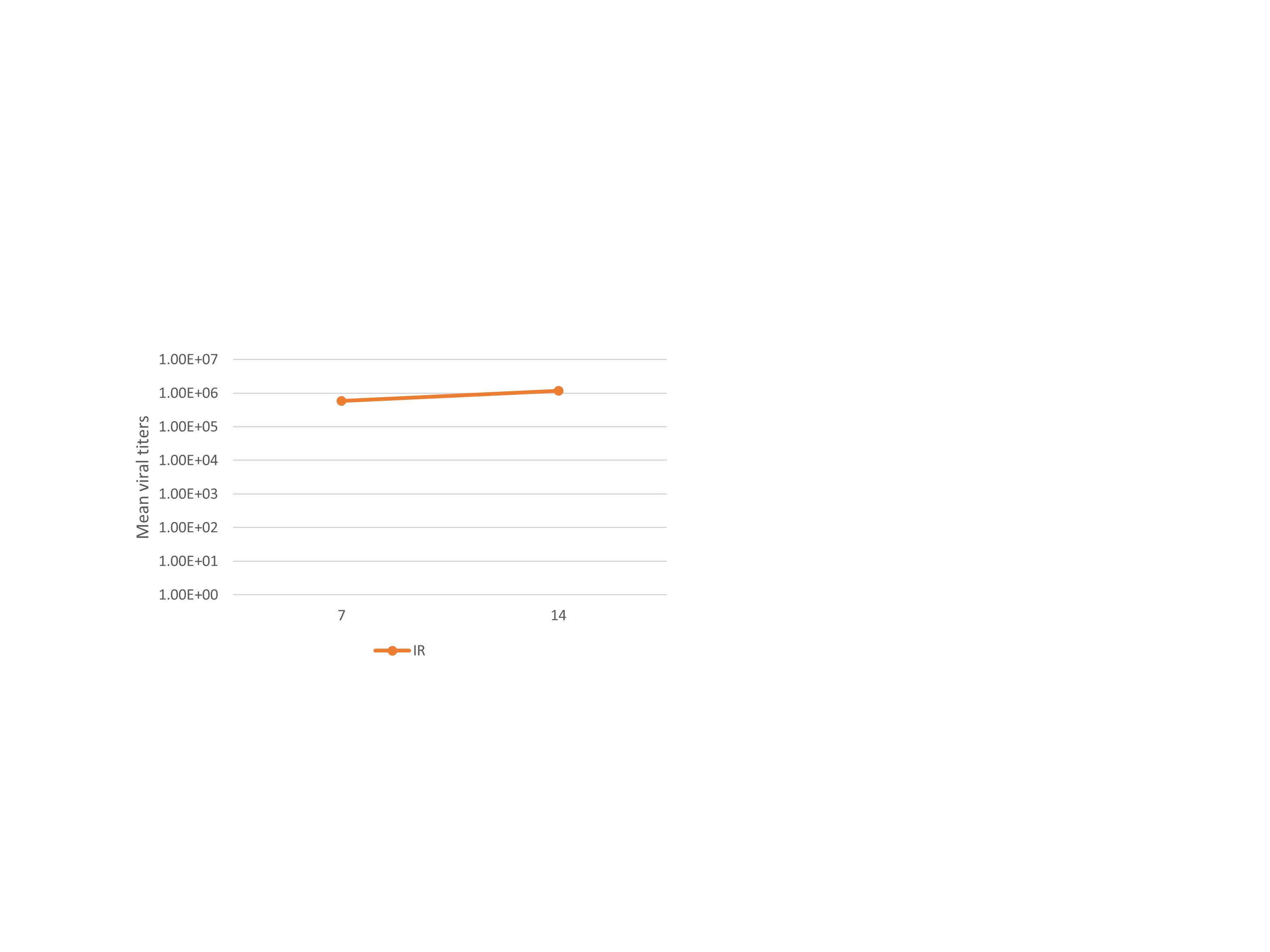

Supplement: S4 Fig — (TIF) [file pntd.0008986.s004.TIF]
